# Note on Hydro-Bromate of Hyoscine

**Published:** 1885-12-20

**Authors:** H. C. Wood


### NOTE ON HYDRO-BROMATE OF HYOSCINE.

BY H. C. WOOD, M.D.

Since my last communication to the Gazette I have used hyoscine in a number of cases with confirmatory results to those previously reached, and at the same time a more complete outlining of the limits of the usefulness of the drug. The dose by the mouth, which is necessary, is somewhat larger than I had at first supposed, most persons not being distinctly affected unless by 1–80 of a grain. A new field of usefulness seems to be in cases of spermatorrhœa, and I have only had the opportunity of using it in three or four cases; but the results have been uniform, and much more correct and assured than those I have ever seen reached by any other drug. Thus, in one case in which the discharges averaged about three a week, they were completely arrested by the exhibition of 1–75 of a grain every night. Unfortunately, the effect is not at all permanent: so soon as the drug is intermitted the tendency appears to recur. Whether the long-continued use of the drug will have a lasting effect or not I am unable at present to say. Indeed, the number of cases which I have myself seen is too few to warrant a positive conclusion; but at my suggestion, Dr. Robinson, physician to the Eastern Penitentiary, Philadelphia, has been testing the drug on the convicts, the disease being exceedingly frequent among them. He informs me that its results have been surprisingly good, and promises a definite statement to the readers of the Gazette in the future. It is interesting here to remark that one of the most successful cases of the use of the drug was seen in a female suffering from erotomaniacal symptoms.

As a soporific the drug is especially successful in those cases in which the insomnia is due to excessive cerebral action—cases in which there is a perpetual and uncontrollable succession of thoughts. Allied to this form of insomnia is the mental condition in which the sleep is excessively disturbed by urgent dreams. This, also, I have seen controlled by hyoscine. In one or two cases of intense fever with delirium the hyoscine has seemed to act very well in controlling the delirium. In two cases the symptoms have been such as to suggest that the drug exerts a paralytic action upon the recurrent laryngeals. One of these cases was in the person of a maniacal female, in whom the hypodermic injection of a large dose of the salt was followed at once by a paroxysm of suffocation apparently paralytic and laryngeal. The second case occurred in a child about ten years old suffering from very violent anginose scarlet fever with moderate laryngeal dyspnœa. Hyoscine was prescribed for the excessive insomnia which has lasted from the onset of the fever, with very good results so far as the insomnia was concerned; but so soon as the child came under the influence of the drug the laryngeal symptoms increased with very great rapidity, and in the course of an hour or two death resulted. Whether the hyoscine was or was not the cause of this increased dyspnœa is of course uncertain; but two cases are assuredly enough to arouse suspicion.—*Therapeutic Gazette.*

